# Generation of human iPSC-derived pancreatic organoids to study pancreas development and disease

**DOI:** 10.12688/f1000research.162496.1

**Published:** 2025-06-10

**Authors:** Jean-Francois Darrigrand, Abigail Isaacson, Francesca Maria Spagnoli

**Affiliations:** 1 Centre for Gene Therapy & Regenerative Medicine, King's College London Faculty of Life Sciences & Medicine, London, England, UK

**Keywords:** organoid differentiation, human induced pluripotent stem cells (hiPSCs), 3D pancreatic model, replacement, branching morphogenesis, lineage specification, acinar fate differentiation, pancreatic diseases.

## Abstract

The pancreas has vital endocrine and exocrine functions that can be affected by life-threatening diseases such as diabetes and pancreatic cancer. Although animal models are essential for understanding pancreatic development and disease, they are limited by their low throughput and major species-specific molecular and physiological differences. Generating 3D *in vitro* models, such as organoids, that are physiologically relevant is essential for investigating pancreatic development and disease in the human context. However, the production of human stem cell-derived pancreatic organoids with a proper branched architecture and correct patterning of cell domains remains challenging. Here, we successfully developed a protocol that efficiently and reproducibly generated organoids from human induced pluripotent stem cells (hiPSCs) by optimizing organoid culture format and media. Our differentiation protocol promotes acinar cell differentiation and generates organoids with branches patterned into the central trunk and peripheral tip domains without relying on animal-derived matrices for organoid culture. This platform opens the door to high-throughput investigations of human pancreatic development in a system that recapitulates the most important aspects of pancreatic tissue architecture. Lastly, we anticipate that this system will contribute to the replacement of animal models used to investigate diseases, such as pancreatic cancer.

Research highlights **Scientific benefits**

• A protocol for the generation of hiPSC-derived pancreatic organoids results in the induction of branching and formation of distinct acinar and ductal cellular domains.
  ○ Branch formation is reproducibly induced in 93.5% of organoids
  ○ AMY+ and NKX6.1+ cells occupy non-overlapping tip and trunk regions.
  ○ Increased physiological relevance was achieved, as evidenced by branching and acinar differentiation, compared to previous hiPSC-derived protocols.
• A high-throughput approach to interrogate cell signalling.

**3Rs benefits**

• The protocol promotes the acinar cell lineage without relying on animal-derived matrices for organoid culture.
• Some animal models are used to study gene function in pancreas development, which often requires large numbers of animals to breed and maintain genotypes of interest.
• Better recapitulation of human pancreas development in organoids than in animal models could result in a shift in model usage for lineage-tracing studies.

**Practical benefits**

• The use of hiPSC lines enables a larger scale of genetic manipulations to be studied and accelerates the timeline for phenotyping compared to mouse experiments.
• The scalability and controllability of organoid models allow more data to be generated via high-throughput experiments, *e.g.* for genetic/drug screens.
• The burden of genotyping animal models, setting up timed matings, and reducing wastage of animal embryos with incorrect genotypes. Once a specific hiPSC line is generated, it can be frozen and reused.

**Current applications**

• An *in vitro* platform for studying pancreatic developmental processes, including cell-cell communication, tissue mechanics, and gene regulation.

**Potential applications**

• Disease modelling, including pancreatitis, Pancreatic ductal adenocarcinoma (PDAC) and diabetes.
• Drug screening purposes.

## Introduction

The pancreas has two vital functions: endocrine and exocrine, which control digestion and blood glucose levels. The exocrine compartment accounts for over 90% of the organ and acinar cells are the primary constituent. ^1^ They are responsible for producing digestive enzymes, such as amylase, protease, and lipase, which are stored as zymogen granules and released by exocytosis during digestion. Additionally, as part of the exocrine compartment, ductal cells form a ramified network of branches that transport digestive enzymes to the duodenum and release bicarbonate ions to neutralize the acidic contents of the stomach.

During embryogenesis, the pancreas originates from the foregut endoderm at embryonic day (E) 9.5, in mice, and 30 days post-conception (dpc) in humans. ^2, 3^ Until E14.5, in mice and 50dpc in human, primary branches form and elongate. ^4, 5^ After E14.5, branches start bifurcating and form a ramified network of ducts connecting all the acini to the duodenum. Concomitant with branch formation, pancreatic cells acquire specialized identities and localize to discrete regions of the developing pancreas. Bipotent progenitor cells located in the trunk regions give rise to ductal and endocrine cells, whereas progenitors in the tip regions give rise to acinar cells. ^6, 7^ Hence, the cellular mechanisms regulating cell differentiation and branching morphogenesis are spatiotemporally synchronized, so that pancreatic cells organize themselves into a branched architecture fundamental for pancreatic function.

Gene regulatory networks (GRNs) governing pancreatic identities have been extensively characterized in mice and are conserved in humans to some extent. ^8– 10^ The transcription factors *PTF1A* and *NKX6.1* respectively govern the allocation of multipotent progenitors to either the acinar fate or ductal/endocrine cell identities. ^9^ However, the roles of many soluble and extracellular matrix proteins in the pancreatic microenvironment remain elusive. The extended time required to study the effects of gene mutations in mice has impeded systematic characterization of developmental signals. Mouse models have been widely employed to study pancreatic development owing to the availability of advanced tools for lineage tracing and gene editing. Although the breeding and maintenance of these models are generally considered mild procedures, they often require large numbers of animals to obtain specific genotypes, collect samples across multiple developmental stages, and ensure experimental reproducibility. Moreover, clear differences have now been described between human and mouse pancreatic development, highlighting the importance of developing human experimental models, ^11^ which would also enable high-throughput functional characterization.

The *in vitro* differentiation of human induced pluripotent stem cells (hiPSCs) followed by cultures in three dimensions (3D) can generate multicellular structures called organoids, which recapitulate key physiological organ features. ^12, 13^ hiPSC-derived organoids enable patient-specific disease modelling and increased genetic diversity compared to non-animal cell sources, such as human embryonic stem cells, for organoid generation. The use of hiPSC-derived organoids enables the systematic targeting of developmental genes using CrispR-Cas9 technology. ^14^ However, most organoid culture protocols rely on animal-derived materials such as Matrigel, which are used as scaffolds to support organoid growth and morphogenesis. Here, we optimized a Matrigel-free 3D culture approach to generate hiPSC-derived pancreatic organoids with branched architecture and proper regionalization of acinar/tip and trunk progenitor compartments.

Understanding what regulates acinar cell differentiation is essential not only for investigating the molecular mechanisms underlying pancreatic development, but also for investigating the underlying pancreatic cell plasticity in disease. Most importantly, acinar cell plasticity is linked to pancreatic inflammation and pancreatic ductal adenocarcinoma (PDAC). During pancreatic inflammation, acinar cells may dedifferentiate into ductal-like progenitor cells that reacquire clear embryonic markers. ^15^ This is a reversible process that allows regeneration of pancreatic tissue after inflammation. However, persistent activation of mutated genes, such as oncogenic *Kras,* leads to permanent dedifferentiation of acinar cells and is linked to PDAC onset in 98% of cases. ^1^ The animal procedures used to model pancreatic inflammation and PDAC range from moderate to severe. ^16, 17^ Besides replacing the use of animal procedures, human pancreatic organoids hold great promise for drug screening purposes. The organoid models developed previously either lacked a physiologically relevant architecture or still required transplantation in mice, and thus remained low-throughput and dependent on animal surgical procedures. ^18^ Hence, we anticipate that the physiological relevance of the pancreatic organoid platform developed in this study could support the development of high-throughput preclinical studies, while replacing mouse models of pancreatic diseases.

## Methods

### hiPSC culture

hiPSCs (AICS-0090-39) ^19^ were obtained from the Coriell Institute for Medical Research (CIRM) and maintained according to the manufacturer’s protocol ( https://www.coriell.org/0/PDF/Allen/ipsc/AICS-0090-391_CofA.pdf) in Matrigel-coated (Corning) 6 well-plates with mTesr1 growth medium (STEMCELL Technologies) supplemented with 1% penicillin-streptomycin (P/S) in a humidified incubator (37°C, 5% CO _2_). hiPSCs were thawed at 37°C in a water bath, and the cell suspension was then transferred dropwise to a Falcon tube containing enough growth medium for a 1:10 dilution. hiPSCs were centrifuged at 300 × RCF for 5 min, resuspended in growth medium containing 5μM Rho-associated protein kinase (ROCK) inhibitor, and then seeded onto Matrigel-coated plates for maintenance.

Cells were passaged as single-cells at 70-80% confluency using Accutase (Invitrogen) and medium supplemented with 5μM ROCK inhibitor Y-27632 (Sigma) on the day of passaging. Passages of hiPSCs exhibiting significant background differentiation were excluded from the differentiation experiments, and hiPSCs were passaged at least once after thawing and prior to commencing differentiation. The number of passages used for all experiments was less than 45. Cryopreservation of hiPSCs was carried out in freezing medium comprising 60% mTesr1, 30% knockout serum replacement (KoSR), and 10% dimethyl sulphoxide for 24h at -80°C using controlled-rate freezing. The cells were then transferred to liquid nitrogen for long-term storage.

### hiPSC differentiation into pancreatic progenitors

hiPSCs were seeded at a density of 200,000-300,000 cells/cm ^2^ in Matrigel-coated 6-well plates and grown for 24h in mTesr1 supplemented with 1% P/S and 5 μM ROCK inhibitor. Cells were subsequently differentiated in a medium that was replenished daily and supplemented with cytokines to induce their differentiation into pancreatic progenitors. The cytokine and small compound concentrations used were as described by Barsby et al. ^20^

Briefly, the first Basal Medium (basal medium 1) used between days (D) 1 and 5 of differentiation was composed of MCDB131 medium supplemented with Glutamax (1x), glucose (2.5 M), NaHCO _3_ (1.5g/l), and BSA (0.5%). The second Basal Medium (basal medium 2) used between D6 and the end of differentiation was made of MCDB131 medium supplemented with Glutamax (1x), glucose (2.5 M), NaHCO _3_ (2.5g/l), BSA (2%), and ITS-X (0.5x). The reagents used are listed in Table 1.

**Table 1.** List of reagents and suppliers.

Reagents	Source
MCDB131 Medium	Gibco (Cat # 10372-019)
Glutamax	Gibco (Cat # 35050-038)
Glucose	Roth (Cat # X997.1)
NaHCO _3_	Gibco (Cat # 11360-039)
BSA	Sigma (Cat # A1470)
ITS-X	Gibco (Cat # 41400-045)
Pen/Strep	Gibco (Cat # 15140122)
Activin A (AA)	Qkine (Cat # QK001)
CHIR-99021 (Chir)	Abcam (Cat # ab120890)
FGF7	Qkine (Cat # QK046)
Vitamin C (Vit. C)	Merck (Cat # A4544)
Sant1	Merck (Cat # S4572)
Retinoic Acid (RA)	Sigma (Cat # R2625)
LDN-193189 (LDN)	Stemgent (Cat # 04-0074)
TPB	Calbiochem (Cat # 565740)
EGF	Qkine (Cat # QK011)
Nicotinamide (Nicot.)	Sigma (Cat # N0636)
ROCK inhibitor Y-27632 (RI)	Sigma (Cat # Y0503)
GSiXX	Millipore (Cat # 565790)
FGF10	Qkine (Cat # QK003)

The supplements used to differentiate the pancreatic progenitors were iteratively added to the basal media, as detailed in Table 2.

**Table 2.** Overview of the supplements and concentrations used to differentiate hiPSCs into pancreatic progenitors.

Days (D)	Basal Medium	Supplements (final concentration)
D1	Basal Medium1	100 ng/ml AA 3.0 μM Chir
D2		100 ng/ml AA 0.3 μM Chir
D3		100 ng/ml AA
D4-D5		50 ng/ml FGF7 0.25 mM Vit. C
D6-D7	Basal Medium 2	50 ng/ml FGF7 0.25 mM Vit. C 0.25 μM Sant1 1 μM RA 100 nM LDN 200 nM TPB
D8-D10		2 ng/ml FGF7 0.25 mM Vit. C 100 ng/ml EGF 0.25 μM Sant1 0.1 μM RA 200 nM LDN 100 nM TPB 10 ng/ml AA 10 mM Nicot. 5 μM RI

### Formation and culture of pancreatic organoids

On D11 of differentiation, pancreatic progenitors (PP) were dissociated using TrypLE (Gibco) and left to aggregate in the Aggrewell microwell system (STEMCELL Technologies) for 72h. Progenitors were seeded in Aggrewell plates at a density of 1200 cells/microwell. After PP clusters had formed in the Aggrewell (72h), they were transferred to suspension plates, and the basal medium was supplemented with the cytokines and small compounds listed in Table 3.

**Table 3.** Overview of the supplements and concentrations used to differentiate pancreatic progenitors into branched pancreatic organoids.

Days (D)	Culture format	Basal Medium	Supplements (final concentration)
D11-D13	AggreWell	Basal Medium 2	2 ng/ml FGF7 0.25 mM Vit. C 100 ng/ml EGF 0.25 μM Sant1 0.1 μM RA 200 nM LDN 100 nM TPB 10 ng/ml AA 10 mM Nicot. 5 μM RI
D14-D15	Suspension		0.25 mM Vit. C 100 ng/ml EGF 0.1 μM RA 100 nM TPB 25 ng/ml FGF7 100 nM GSiXX 10 mM Nicot.
D16-D18	Suspension		25 ng/ml FGF7 0.25 mM Vit. C 100 ng/ml EGF 1 μM RA 100 nM TPB 100 nM GSiXX 10 mM Nicot. 100 ng/ml FGF10 10 ng/ul AA 100 nM LDN

Here, we describe a step-by-step protocol for the generation and culture of pancreatic organoids derived from pancreatic progenitors cultured in 6-well plates and collected on D11 of differentiation. The general steps of cell dissociation, aggregation, and suspension culture were similar to those described by Barsby et al. However, the timing and culture media have been modified and optimized to induce exocrine differentiation and branching morphogenesis, instead of endocrine differentiation. The reagents used in these steps are listed in Table 2.

### Day 11

*Preparation of the Aggrewell microwell system (24-well plate)*

- Add 500 μL of anti-adherence rinsing solution to each well and centrifuge at 1300 RCF for 5 min.
- The solution was removed by aspirating the anti-adherence solution and add 500 μL of MCDB131 medium.

*Dissociation and collection of pancreatic progenitors*

- Wash pancreatic progenitors (cultured in a 6-well plate) by adding 1 ml of versene to each well and removing it after 1 min.
- Add 1ml of TrypLE to each well and incubate for 8min at 37°C.
- Keep at 37°C until mild-tapping of the plate causes cell detachment.
  Add 1 ml of MCDB131 medium per well and gently flush until most cells are detached.
  Add another 1 ml of MCDB131 medium per well and collect cells from all wells into a 50 ml tube.
- Count cells
  Centrifuge the 50 ml tube with the cell suspension at 300 RCF for 3 min.
- Aspirate the supernatant and gently resuspend the cells in pre-warmed Basal Medium 2 supplemented with cytokines and small compounds listed in Table 3 (D11-D13).
- MCDB131 medium was aspirated from the Aggrewell plate, replace with 2mL D11-D13 medium, and pancreatic progenitors were seeded at a density of 1200 cells/microwell.
- Centrifuge the plate at 100 g for 3 min.
- Incubate plates in a humidified cell culture incubator (37°C, 5%CO _2_).

### Day 12-13

*Media changes*

- Carefully remove 1 ml of medium from each well by keeping the plate horizontal and aspirating slowly with a pipette tip while taking care not to dislodge clusters at the base of the well.

Add 1 ml of fresh pre-warmed D11-D13 medium gently on the top side of the wells.

### Day 14

*Prepare D14 culture medium*

On this day, cell clusters collected from 2 wells of the Aggrewell plate are pooled in one ultra-low attachment (ULA) well of a 6-well plate.

- Prepare enough D14-D15 medium (Basal Medium 2 with supplements listed in Table 3) to add 5 ml per well of the ULA 6-well plate.

*Transfer of pancreatic progenitor clusters to suspension culture*

- For each well of the Aggrewell plate, 1 ml of medium and gently flushed out from the edge of the well to dislodge cell clusters from the microwells.
  Coat the pipette tip used to transfer the clusters to the medium.
  Swirl the Aggrewell plate to pool the cell clusters toward the center of the well and transfer them into a ULA plate. Pool cell clusters were collected from two wells of the Aggrewell plate in one ULA well.
  Once all cell clusters have been transferred into the ultra-low attachment plate, swirl this plate gently in one direction to pool the clusters into the center.
- Remove most of the medium by ensuring that cell clusters are not aspirated, which should be visually visible.
- Add 5ml of D14-D15 medium per well.
- Place the plate in a humidified cell incubator (37°C, 5% CO _2_) on a rotating platform (100RPM).

### Day 15

- Swirl the ULA plate to center cell clusters in the wells.
- Remove 4 ml of medium by aspirating from the well edge and replacing it with fresh D14-D15 medium.
- Place the plate back into a humidified cell incubator (37°C, 5% CO _2_) on a rotating platform (100RPM).

### Day 16-18

- Swirl the ULA plate to center cell clusters in the wells.
- Check the formation of branches as a marker for efficient organoid maturation.
- Remove 4 ml of medium by aspirating from the well edge and replacing it with fresh D16-D18 medium.
- Place the plate back into a humidified cell incubator (37°C, 5% CO _2_) on a rotating platform (100RPM).

### Day 19

Experiments were performed using pancreatic organoids or following the steps below to fix the organoids.

- Swirl the ULA plate to center the organoids in the wells.
- Collect and pool organoids in a tube.
- Centrifuge at 200 g for 2 min.
- Wash in PBS and centrifuge again before fixing in 4 % paraformaldehyde for 30 min at 4°C.
- Keep in PBS at 4°C.

### Immunofluorescence and RT-qPCR

*Immunostaining of cells*

In addition to the 6-well plates used to generate pancreatic progenitor-derived organoids, hiPSCs were also cultured in 24-well plates in 1 ml differentiation medium at a cell seeding density of 200,000-300,000 cells/cm ^2^. These plates were used to confirm the efficiency of hiPSC differentiation into pancreatic progenitors by immunostaining for acinar and ductal progenitor markers at D14. Cells were washed in 1ml PBS, fixed for 20 min at 4°C with 4 % paraformaldehyde, and washed again. Fixed cells were then blocked for 45min in a blocking buffer composed of PBS, Donkey serum (Sigma) (3%), and Triton-X (0.1%). Primary antibodies were added to the blocking buffer at dilutions listed in Table 4 and incubated overnight at 4°C. The next day, cells were washed multiple times in PBS with Triton-X (0.1%) and incubated for 1 h in the dark with the secondary antibodies diluted in blocking buffer ( Table 4) and counterstained with Hoechst. Before imaging, the cells were washed multiple times with PBS.

**Table 4.** List of reagents to perform immunostainings of cells and organoids.

Reagent	Source
Guinea pig anti-PDX1 (1:300)	Abcam (Cat # AB47308; RRID: AB_777178)
Mouse anti-NKX6.1 (1:400)	DSHB (Cat #F55A10; RRID: AB_532378)
Rabbit anti-AMYLASE (1:400)	Merck (Cat #A8273; RRID: AB_258380)
Hoechst 33342 (250 ng/mL)	Invitrogen (Cat #H1399)

*Immunostaining of organoids*

Throughout the staining procedure, the solutions were changed by centrifuging the organoids at 200 g for 2 min, removing the supernatant, and adding the next solution.

First, fixed organoids were blocked for 24h in a blocking buffer made from PBS, Donkey serum (3%), and Triton-X (0.5%) at 4°C on a rotating platform. Primary antibodies were added to the blocking buffer at the dilutions listed in Table 4 and incubated for 24h under similar conditions. The next day, organoids were washed three times for 45min in PBS with Triton-X (0.1%), and then incubated for 24h in the dark at 4°C on a rotating platform with the secondary antibodies diluted in blocking buffer ( Table 4), and counterstained with Hoechst. Before imaging, organoids were washed twice in PBS with Triton-X (0.1%) for 45min and once in PBS.

*Imaging and image analysis*

Immunostained organoids were placed in a 96-well plate with a glass bottom and imaged using a Zeiss LSM 700 confocal microscope. Using ImageJ, ^21^ we quantified cell numbers using the in-built cell counter tool and measured PDX1 staining intensity levels in the nuclei by creating a mask on the Hoechst channel. PDX1 intensity was quantified by measuring the mean pixel intensity in the region of interest (ROI) delineating the AMY+ and NKX6.1+ cells.

*RT-qPCR*

Cells from the planar culture were dissociated and harvested with TrypLE, and organoids/cell clusters were collected with a trimmed pipette tip pre-coated with 1mL of medium previously removed from the well. These were centrifuged for 3 min at 300 RCF or for 2 min at 200 RCF, respectively. The supernatant was aspirated, and the pellets were washed once with PBS (and then frozen at -80°C). A High Pure RNA Isolation Kit (Roche) was used for RNA extraction and all samples were treated with DNase to remove genomic DNA. cDNA was synthesized using a Transcriptor First Strand cDNA Synthesis Kit (Roche). RT-qPCR was performed on a LightCycler (Roche) with FastStart Essential SYBR Green Master Mix (Roche). The 2-ΔΔCt method ^22^ was used to evaluate the relative gene expression, and the housekeeping gene glyceraldehyde-3-phosphate dehydrogenase (GAPDH) was used for normalization. The cDNA dilution was modified to return Cts of approximately 20 for GAPDH, and missing Ct values were set to 40 when the threshold for detectable fluorescence was not met. The primers used for RT-qPCR are listed in Table 5.

**Table 5.** List of primers used to perform RT-qPCR on hiPSC-derived cells.

Primer	Sequence
GAPDH F	5’ TGC ACC ACC AAC TGC TTA GC 3’
GAPDH R	5’ GGC ATG GAC TGT GGT CAT GAG 3’
NKX6.1 F	5’ TGG CCT ATT CGT TGG GGA TG 3’
NKX6.1 R	5’ TGT CTC CGA GTC CTG CTT CT 3’
PDX1 F	5’ CAC ATC CCT GCC CTC CTA C 3’
PDX1 R	5’ GAA GAG CCG GCT TCT CTA AAC 3’
AMY F	5’ TAC CGT TGG CCA AGA CAG TT 3’
AMY R	5’ TCA CAG ACC CAG TCA TTG CC 3’
CPA1 F	5’ GT AAG CGT CCA GCC ATC TG 3’
CPA1 R	5’ TGT CGA GAA TGG CGG TGA AA 3’

*Quantification and statistical analysis*

All statistical analyses were performed using the R package rstatix. The elements shown in the boxplots are the 25% (Q1, upper box boundary), 50% (median, black line within the boxes), and 75% (Q3, lower box boundary) quartiles, and the whiskers represent a maximum of 1.5X × interquartile range. P-values were calculated using a two-tailed t-test. Normality was assessed using the Shapiro-Wilk normality test. Statistical significance was calculated using unpaired Student’s t-tests for data with normal distributions, and Mann-Whitney U-tests otherwise.

## Results

### Generation of pancreatic progenitors

**Figure 1.**
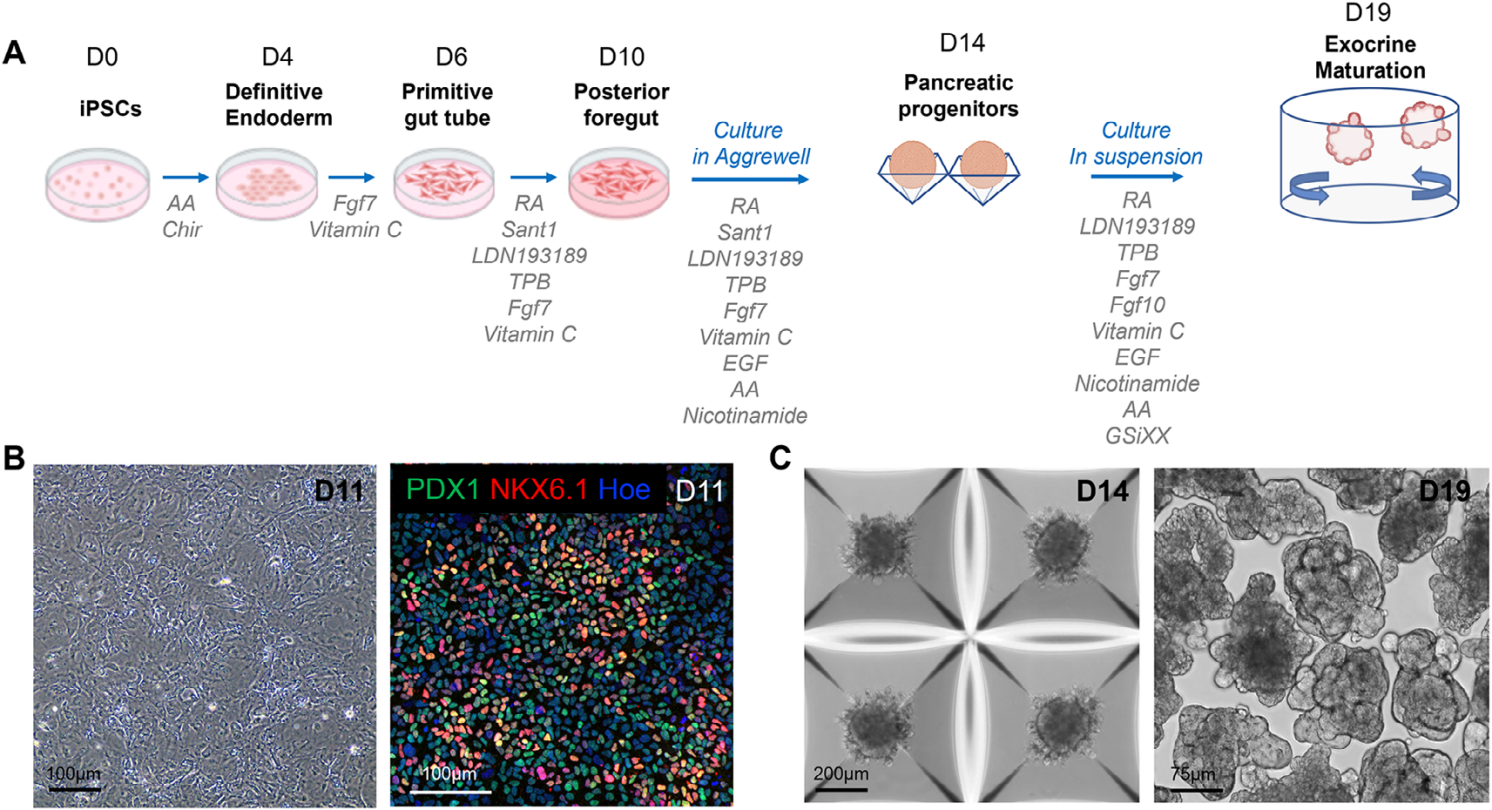
Differentiation of hiPSCs into branched pancreatic organoids. A. Schematic overview and media composition for the differentiation of hiPSCs into pancreatic organoids. The differentiation was carried out as a monolayer until day 11 (D11), then transferred to the Aggrewell microwell system from D12 to D14, and finally performed in suspension until D19. B. Representative phase contrast image (left panel) and confocal image (right panel) of D11 pancreatic progenitors immunostained with the PDX1 and NKX6.1 pancreatic markers. C. Representative phase contrast images of pancreatic clusters cultured in the Aggrewell microwell system on D14 of the differentiation (left panel) and in suspension on D19 of the differentiation (right panel). AA: Activin A, RA: Retinoic Acid.

hiPSCs were differentiated into pancreatic progenitors as described by Barsby et al. ^20^ with minor modifications, which are outlined in the Materials and Methods ( Figure 1A). The efficiency of pancreatic progenitor differentiation was monitored by assessing the morphology of differentiating cells with a phase contrast light microscope between day (D8) and D10 of differentiation and by immunostaining for the pancreatic progenitor markers PDX1 and NKX6.1, at D11 of differentiation ( Figure 1B). Pancreatic progenitors were predominantly double-positive for PDX1 and NKX6.1, and clusters were efficiently formed within 24 h in the Aggrewell plate.

### Generation of branched organoids

**Figure 2.**
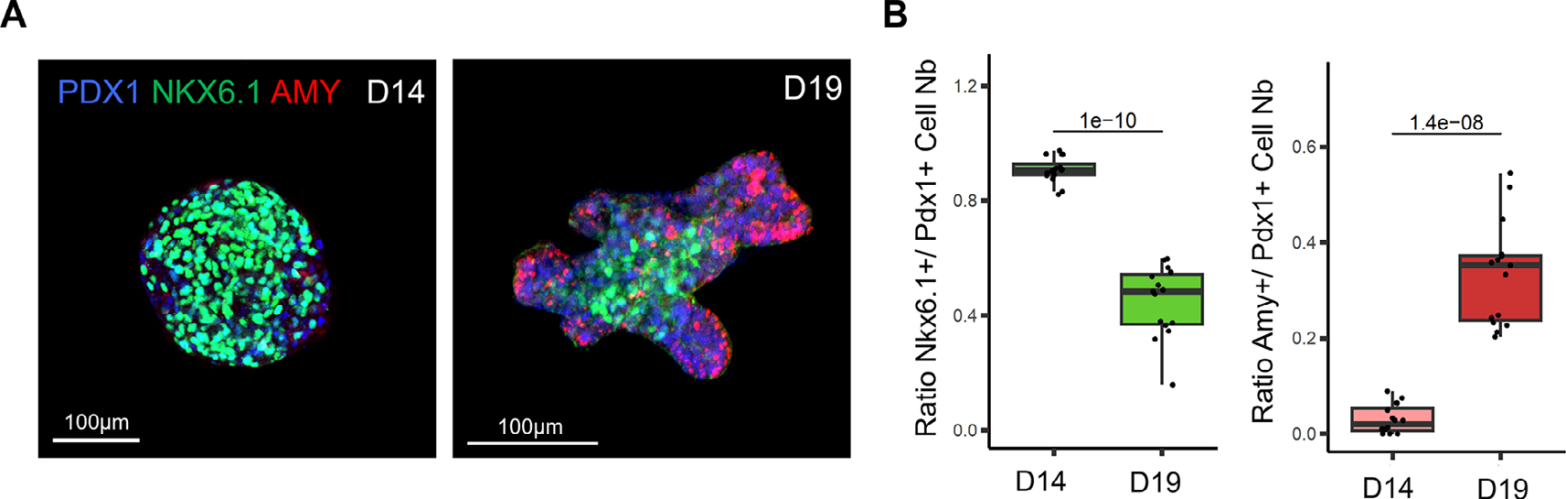
Physiological patterning of the pancreatic organoid branches. A. Representative confocal images of D14 pancreatic clusters (left panel) and D19 pancreatic organoids (right panel) immunostained for PDX1, NKX6.1 and Amylase (AMY), which respectively mark all pancreatic progenitor cells, trunk progenitor cells and acinar cells. B. Ratio of PDX1+ cells positive for the trunk marker NKX6.1 at D14 and D19 (left panel). Ratio of PDX1+ cells positive for acinar marker Amy at D14 and D19 (right panel). N=13-15 organoids per stage analysed, from N=3 independent differentiation experiments. P-values generated from two-tailed t-tests are displayed on the boxplots.

The dissociated pancreatic progenitors were left to aggregate in Aggrewell plates ( Figure 1C). After progenitor clusters were formed, they were transferred to suspension plates for the final stage of differentiation, using an optimized differentiation medium (see composition in Table 3). Specifically, we supplemented the medium with GSiXX, a Notch inhibitor known to promote pancreatic cell differentiation, ^23, 24^ along with FGF7 and FGF10, which stimulate the growth and morphogenesis of the exocrine pancreas. ^25^ In contrast to Barsby et al., ^20^ we excluded factors known to induce endocrine differentiation, such as thyroid hormone receptor agonists, TGF-β antagonists, and betacellulin. This medium, combined with the suspension culture format, triggered primary branch formation in organoids between D17 and D18. 93.5% (SD = 2.3) of organoids acquired a branched architecture by D19 of the differentiation, highlighting a high efficiency of morphogenesis ( Figure 1C; 2A).

To investigate the efficiency of our culture medium to induce exocrine differentiation in organoids, we performed immunostaining for tip and trunk markers ( Figure 2A). Our whole-mount immunostaining highlights that the stalk region of the organoid branches is positively marked by the NKX6.1 trunk marker, whereas the tip regions are marked by the amylase acinar marker. Hence, the pattern of expression of both these markers is mutually exclusive and clearly defines the tip and trunk domains in organoids. In addition, our quantification showed that the acinar domain strongly increased in the last stage of differentiation between D14 and D19, demonstrating that our protocol supports acinar cell differentiation ( Figure 2B). Altogether, our differentiation protocol recapitulated the patterning of the pancreatic branches observed in vivo, with acinar cells positioned at the tip of the branches.

### Analysis of acinar cell differentiation

**Figure 3.**
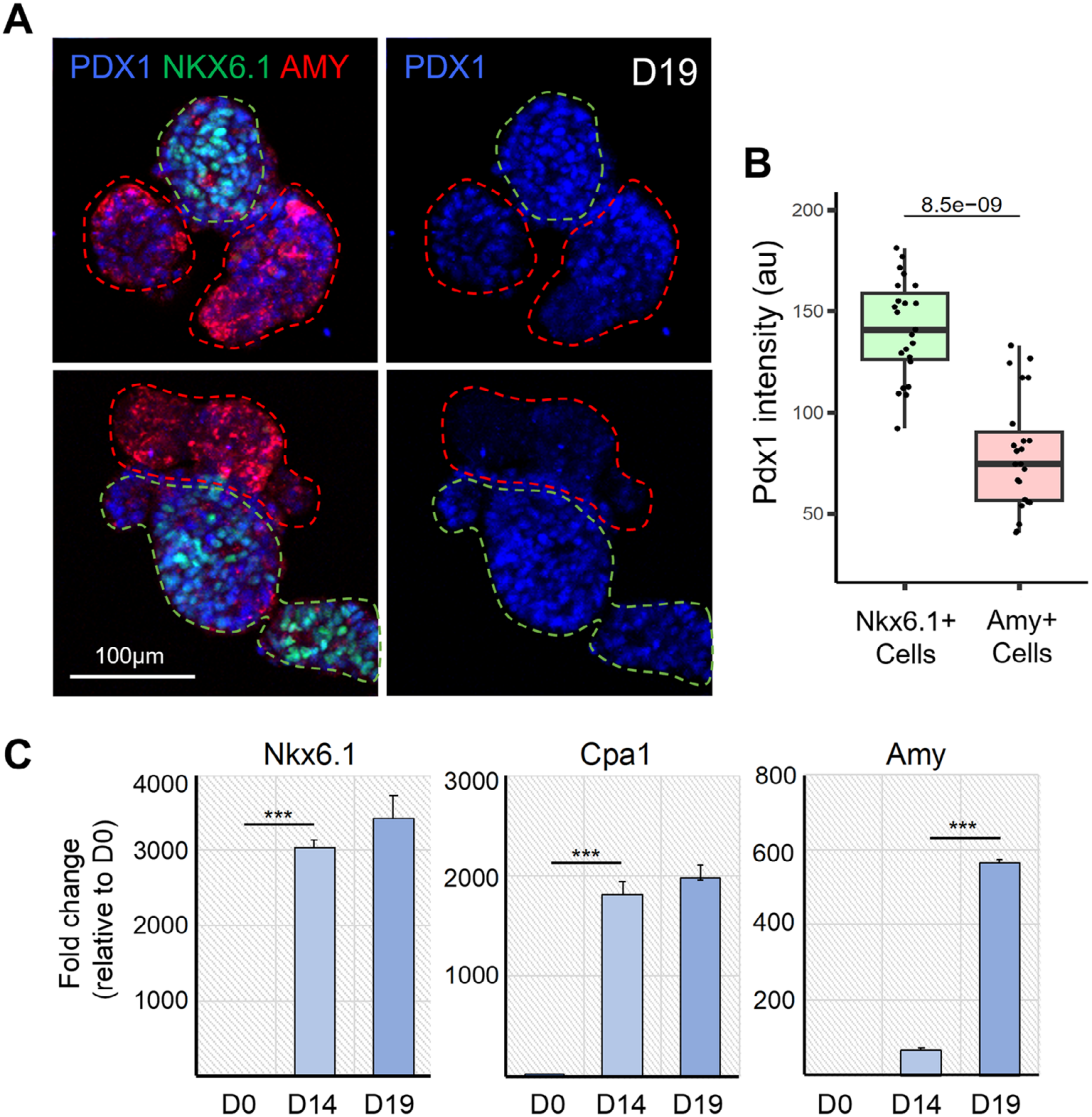
Differentiation of acinar cells in branched pancreatic organoids. A. Representative confocal images of D19 pancreatic organoids immunostained for PDX1, NKX6.1 and AMY, which respectively mark all pancreatic progenitor cells, trunk bipotent cells and acinar cells (left panel). PDX1 is shown alone in the right panel. Dotted green and red lines respectively delineate the NKX6.1+ trunk and AMY+ tip regions. B. Quantification of PDX1 staining intensity in the NKX6.1+ and AMY+ cells. N = 17-22 organoids analysed, from N = 3 independent differentiation experiments. The p-value generated from two-tailed t-test is displayed on the boxplot. C. Representative qRT-PCR analysis (N = 1 shown) for the indicated pancreatic genes performed on cells, clusters and organoids collected at D0, D14 and D19 of the differentiation protocol. Data are represented as relative fold change. Values shown are mean ± SD. ∗p < 0.05, ∗∗p < 0.01, ∗∗∗p < 0.001; two-tailed paired t tests.

It has been previously shown that during in vivo pancreatic development, PDX1 expression levels decrease in differentiating acinar cells. ^26^ To confirm that this process was recapitulated in our organoid system, we quantified PDX1 staining intensity in AMY+ acinar cells compared to NKX6.1+ cells in organoids collected on D19 ( Figure 3A). Our results showed that the PDX1 staining intensity was significantly lower in AMY+ acinar cells than in NKX6.1+ cells at this stage, thus recapitulating the decrease in PDX1 expression observed in vivo.

In addition, we performed RT-qPCR on dissociated cells collected at D0, D14, and D19 to investigate the efficiency of our protocol in inducing cell differentiation, specifically the acinar fate. Our results highlight that differentiating cells acquire a pancreatic fate by D14, as evidenced by the strong expression of the pancreatic progenitor markers *NKX6.1* and *CPA1* ( Figure 3B). Between D14 and D19, we observed that the level of *Amylase* expression was strongly increased in organoids, and *CPA1* expression was also maintained, indicating that our organoid culture medium promoted the differentiation of acinar cells. Altogether, the differentiation of acinar cells and the formation of a branched architecture demonstrated that our protocol enables the formation of pancreatic organoids with an exocrine identity.

## Conclusions/Discussion

The derivation of pancreatic organoids from either stem cells or primary tissue allows the possibility of replicating native pancreatic organization, morphology, and the emergence of distinct cell populations in health and disease. ^27^ The cell sources of human pancreatic organoids include hPSCs, primary cells from adult tissues, and fetal tissue obtained between 7-11 wpc. ^13^ Human PSC-derived pancreatic organoids are capable of giving rise to ductal or acinar cells that usually self-organize as polarized spheres. ^28, 29^ However, the generation of human stem cell-derived pancreatic organoids with a proper branched architecture and correct patterning of the acinar/tip and trunk cell domains has been an ongoing challenge.

The advent of pancreatic organoids for use as an *in vitro* model has followed many years of optimization of pancreatic cell differentiation protocols, ^30– 32^ which aim to mimic pancreatic development. Most recently, a highly optimized protocol was developed ^20^ that incorporated several existing protocols and specifically used SHH and BMP inhibition, FGF and RA signalling activation, and the discovery that joint administration of Nicotinamide and Epidermal Growth Factor (EGF) can increase pancreatic progenitor cell yield. ^33^ Nicotinamide has been shown to increase the proportion of PDX1+NKX6.1+ cells by inhibiting CK1 and ROCK, ^34^ while EGF is known to activate the MAP kinase pathway and is required for pancreatic progenitor proliferation. ^35^ This combination resulted in a significantly improved yield of PDX1+NKX6.1+ pancreatic progenitors compared to established protocols. ^20, 36^ Incorporation of this protocol was essential for our work, as it has also been reported that a differentiation efficiency resulting in fewer than 60% PDX1+NKX6.1+ pancreatic progenitors typically does not form aggregates of sufficient size when using the Aggrewell platform. This result also highlights the importance of adequately replicating the in vivo complexity to achieve effective production of pancreatic cell types with high fidelity.

Here, we present a protocol for the generation of hiPSC-derived pancreatic organoids that resemble the neonatal pancreas. We have shown that these organoids harbor regionalized tip and trunk domains. Prominent branching and organoid expansion were observed during the final stage of differentiation, which may have resulted from supplementation with Fgf7 and Fgf10, which are important for the growth and morphogenesis of the exocrine pancreas. ^37, 38^ Fgf10 is also known to interact with and activate Notch to maintain pancreatic progenitor cell self-renewal. ^38– 40^ However, Notch suppression is required for proper lineage diversification and specification, ^23, 41^ and several protocols use media supplemented with Notch inhibitors for the differentiation of PSCs into different pancreatic cell types *in vitro.* ^31, 42, 43^ Since in this study, Notch inhibitor was applied from D14 onwards, our findings suggest that regionalization is at least in part achieved by D14, after which Notch inhibition enables regionalized cell differentiation. The use of the Aggrewell microwell platform was a necessary intermediary step in creating an environment that facilitated cell interactions in 3D, without which branched organoid structures could not be generated. Moreover, our protocol eliminates the need for matrices such as Matrigel or collagen to culture pancreatic organoids. This is a significant advantage, as it reduces the use of animal-derived reagents and minimizes the unpredictability of organoid differentiation efficiency due to reagent batch-to-batch variability.

Further optimization will be needed to determine whether the maturation of these organoids can be improved when cultured for extended periods of time and if they can undergo directed differentiation into endocrine cell types. An *in vitro* model that replicates pancreatic morphology and harbors all pancreatic cell types would equip scientists to answer questions on how branching morphogenesis is coupled with cell differentiation and the role of neighboring and rare cell types during human pancreas development. Endocrine cell differentiation in these organoids would also facilitate research on monogenic forms of diabetes and type 1 and 2 diabetes (T1/2D) using patient-derived cell lines. In the case of T1D, a co-culture system consisting of patient-derived pancreatic organoids and immune cells could also provide a means to study patient-specific beta-immune cell interactions.

The fact that pancreatic cells dedifferentiate into embryonic-like states during pancreatic inflammation and PDAC, ^15^ which are the states replicated in our system, suggests that our organoid platform could allow *in vitro* modelling of many aspects of these diseases and enable preclinical investigations in a high-throughput setup while replacing animal models. Pancreatic organoids derived from pluripotent human stem cells have been used to study PDAC initiation and progression in culture by induction of KRAS and GNAS oncogenes. ^29, 44^ To date, these studies have been performed using cystic organoids composed solely of ductal or acinar cells. Inducing the expression of KRAS and GNAS oncogenes in our organoid platform would enable the research community to study the effects of these mutations in a system that recapitulates the branched architecture of the pancreas and the patterning of its acinar and ductal compartments. Moreover, the high-throughput nature of this platform could allow the screening of drugs that reduce tissue alterations caused by oncogene induction, and thus identify potential therapeutic targets for pancreatitis and PDAC.

## Ethical considerations

Ethical approval and consent were not required,

## Data Availability

Figshare: Generation of human iPSC-derived pancreatic organoids to study pancreas development and disease, https://doi.org/10.6084/m9.figshare.2855930 ^45^ This project contains the following underlying data:
1. Confocal image of pancreatic progenitors immunostained with the PDX1 and NKX6.1 2. Phase contrast image of pancreatic clusters cultured in Aggrewell microwell 3. Phase contrast image of pancreatic clusters cultured in suspension 4. Phase contrast image of pancreatic progenitors 5. Counts of PDX1+ cells positive for the trunk marker NKX6.1 and for acinar marker Amylase 6. Image of pancreatic clusters stained for NKX6.1 and Amylase 7. Image of pancreatic organoid stained for NKX6.1 and Amylase 8. Confocal image of pancreatic organoid immunostained for PDX1, NKX6.1 and AMY 1 9. Confocal image of pancreatic organoid immunostained for PDX1, NKX6.1 and AMY 2 10. Measures of PDX1 Intensity in AMY+ and NKX6.1+ cells v2 11. qPCR data Nkx6.1, CPA1, AMY - organoid differentiation D0 D14 D19 Data are available under the terms of the Creative Commons Attribution 4.0 International license (CC-BY 4.0). The pre-print version of this article has been deposited in BioRxiv at https://doi.org/10.1101/2024.10.24.620102.

## References

[1] Marstrand-Daucé L : Acinar-to-Ductal Metaplasia (ADM): On the Road to Pancreatic Intraepithelial Neoplasia (PanIN) and Pancreatic Cancer. *Int. J. Mol. Sci.* 2023;24:9946. 10.3390/ijms24129946 37373094 PMC10298625

[2] Pan FC Wright C : Pancreas organogenesis: From bud to plexus to gland. *Dev. Dyn. Off. Publ. Am. Assoc. Anat.* 2011;240:530–565. 10.1002/dvdy.22584 21337462

[3] Nair G Hebrok M : Islet formation in mice and men: Lessons for the generation of functional insulin-producing β-cells from human pluripotent stem cells. *Curr. Opin. Genet. Dev.* 2015;32:171–180. 10.1016/j.gde.2015.03.004 25909383 PMC4523641

[4] Villasenor A Chong DC Henkemeyer M : Epithelial dynamics of pancreatic branching morphogenesis. *Dev. Camb. Engl.* 2010;137:4295–4305. 10.1242/dev.052993 PMC2990215 21098570

[5] Shih HP Wang A Sander M : Pancreas organogenesis: From lineage determination to morphogenesis. *Annu. Rev. Cell Dev. Biol.* 2013;29:81–105. 10.1146/annurev-cellbio-101512-122405 23909279

[6] Larsen HL Grapin-Botton A : The molecular and morphogenetic basis of pancreas organogenesis. *Semin. Cell Dev. Biol.* 2017;66:51–68. 10.1016/j.semcdb.2017.01.005 28089869

[7] Zhou Q : A multipotent progenitor domain guides pancreatic organogenesis. *Dev. Cell.* 2007;13:103–114. 10.1016/j.devcel.2007.06.001 17609113

[8] Petersen MBK Gonçalves CAC Kim YH : Recapitulating and Deciphering Human Pancreas Development From Human Pluripotent Stem Cells in a Dish. *Curr. Top. Dev. Biol.* 2018;129:143–190. 10.1016/bs.ctdb.2018.02.009 29801529

[9] Schaffer AE Freude KK Nelson SB : Nkx6 transcription factors and Ptf1a function as antagonistic lineage determinants in multipotent pancreatic progenitors. *Dev. Cell.* 2010;18:1022–1029. 10.1016/j.devcel.2010.05.015 20627083 PMC3133668

[10] Ma Z : Deciphering early human pancreas development at the single-cell level. *Nat. Commun.* 2023;14:5354. 10.1038/s41467-023-40893-8 37660175 PMC10475098

[11] Jennings RE Berry AA Strutt JP : Human pancreas development. *Dev. Camb. Engl.* 2015;142:3126–3137. 10.1242/dev.120063 26395141

[12] Kim J Koo B-K Knoblich JA : Human organoids: Model systems for human biology and medicine. *Nat. Rev. Mol. Cell Biol.* 2020;21:571–584. 10.1038/s41580-020-0259-3 32636524 PMC7339799

[13] Grapin-Botton A Kim YH : Pancreas organoid models of development and regeneration. *Dev. Camb. Engl.* 2022;149:dev201004. 10.1242/dev.201004 36314540

[14] Gopal S Rodrigues AL Dordick JS : Exploiting CRISPR Cas9 in Three-Dimensional Stem Cell Cultures to Model Disease. *Front. Bioeng. Biotechnol.* 2020;8:692. 10.3389/fbioe.2020.00692 32671050 PMC7326781

[15] Jensen JN : Recapitulation of elements of embryonic development in adult mouse pancreatic regeneration. *Gastroenterology.* 2005;128:728–741. 10.1053/j.gastro.2004.12.008 15765408

[16] Hyun JJ Lee HS : Experimental models of pancreatitis. *Clin. Endosc.* 2014;47:212–216. 10.5946/ce.2014.47.3.212 24944983 PMC4058537

[17] Gopinathan A Morton JP Jodrell DI : GEMMs as preclinical models for testing pancreatic cancer therapies. *Dis. Model. Mech.* 2015;8:1185–1200. 10.1242/dmm.021055 26438692 PMC4610236

[18] Boj SF : Organoid models of human and mouse ductal pancreatic cancer. *Cell.* 2015;160:324–338. 10.1016/j.cell.2014.12.021 25557080 PMC4334572

[19] Tian R : CRISPR Interference-Based Platform for Multimodal Genetic Screens in Human iPSC-Derived Neurons. *Neuron.* 2019;104:239–255.e12. 10.1016/j.neuron.2019.07.014 31422865 PMC6813890

[20] Barsby T : Differentiating functional human islet-like aggregates from pluripotent stem cells. *STAR Protoc.* 2022;3:101711. 10.1016/j.xpro.2022.101711 36136756 PMC9508476

[21] Abràmoff MD : Image Processing with ImageJ.

[22] Livak KJ Schmittgen TD : Analysis of relative gene expression data using real-time quantitative PCR and the 2(-Delta Delta C(T)) Method. *Methods San Diego Calif.* 2001;25:402–408. 10.1006/meth.2001.1262 11846609

[23] Hald J : Activated Notch1 prevents differentiation of pancreatic acinar cells and attenuate endocrine development. *Dev. Biol.* 2003;260:426–437. 10.1016/S0012-1606(03)00326-9 12921743

[24] Esni F : Notch inhibits Ptf1 function and acinar cell differentiation in developing mouse and zebrafish pancreas. *Development.* 2004;131:4213–4224. 10.1242/dev.01280 15280211

[25] Miralles F Czernichow P Ozaki K : Signaling through fibroblast growth factor receptor 2b plays a key role in the development of the exocrine pancreas. *Proc. Natl. Acad. Sci. USA.* 1999;96:6267–6272. 10.1073/pnas.96.11.6267 10339576 PMC26870

[26] Miyatsuka T : Persistent expression of PDX-1 in the pancreas causes acinar-to-ductal metaplasia through Stat3 activation. *Genes Dev.* 2006;20:1435–1440. 10.1101/gad.1412806 16751181 PMC1475756

[27] Hohwieler M Müller M Frappart P-O : Pancreatic Progenitors and Organoids as a Prerequisite to Model Pancreatic Diseases and Cancer. *Stem Cells Int.* 2019;2019:9301382. 30930950 10.1155/2019/9301382 PMC6410438

[28] Bakhti M : Establishment of a high-resolution 3D modeling system for studying pancreatic epithelial cell biology in vitro. *Mol. Metab.* 2019;30:16–29. 10.1016/j.molmet.2019.09.005 31767167 PMC6812400

[29] Huang L : Commitment and oncogene-induced plasticity of human stem cell-derived pancreatic acinar and ductal organoids. *Cell Stem Cell.* 2021;28:1090–1104.e6. 10.1016/j.stem.2021.03.022 33915081 PMC8202734

[30] Nostro MC : Stage-specific signaling through TGFβ family members and WNT regulates patterning and pancreatic specification of human pluripotent stem cells. *Dev. Camb. Engl.* 2011;138:861–871. 10.1242/dev.055236 PMC3035090 21270052

[31] Rezania A : Reversal of diabetes with insulin-producing cells derived in vitro from human pluripotent stem cells. *Nat. Biotechnol.* 2014;32:1121–1133. 10.1038/nbt.3033 25211370

[32] Russ HA : Controlled induction of human pancreatic progenitors produces functional beta-like cells in vitro. *EMBO J.* 2015;34:1759–1772. 10.15252/embj.201591058 25908839 PMC4516429

[33] Nostro MC : Efficient Generation of NKX6-1+ Pancreatic Progenitors from Multiple Human Pluripotent Stem Cell Lines. *Stem Cell Rep.* 2015;4:591–604. 10.1016/j.stemcr.2015.02.017 25843049 PMC4400642

[34] Zhang Y : Nicotinamide promotes pancreatic differentiation through the dual inhibition of CK1 and ROCK kinases in human embryonic stem cells. *Stem Cell Res Ther.* 2021;12:362. 10.1186/s13287-021-02426-2 34172095 PMC8235863

[35] Bonfanti P : Ex Vivo Expansion and Differentiation of Human and Mouse Fetal Pancreatic Progenitors Are Modulated by Epidermal Growth Factor. *Stem Cells Dev.* 2015;24:1766–1778. 10.1089/scd.2014.0550 25925840

[36] Balboa D : Functional, metabolic and transcriptional maturation of human pancreatic islets derived from stem cells. *Nat. Biotechnol.* 2022;40:1042–1055. 10.1038/s41587-022-01219-z 35241836 PMC9287162

[37] Bhushan A : Fgf10 is essential for maintaining the proliferative capacity of epithelial progenitor cells during early pancreatic organogenesis. *Dev. Camb. Engl.* 2001;128:5109–5117. 10.1242/dev.128.24.5109 11748146

[38] Miralles F Lamotte L Couton D : Interplay between FGF10 and Notch signalling is required for the self-renewal of pancreatic progenitors. *Int. J. Dev. Biol.* 2006;50:17–26. 10.1387/ijdb.052080fm 16323074

[39] Norgaard GA Jensen JN Jensen J : FGF10 signaling maintains the pancreatic progenitor cell state revealing a novel role of Notch in organ development. *Dev. Biol.* 2003;264:323–338. 10.1016/j.ydbio.2003.08.013 14651921

[40] Hart A Papadopoulou S Edlund H : Fgf10 maintains notch activation, stimulates proliferation, and blocks differentiation of pancreatic epithelial cells. *Dev. Dyn. Off. Publ. Am. Assoc. Anat.* 2003;228:185–193. 10.1002/dvdy.10368 14517990

[41] Murtaugh LC Stanger BZ Kwan KM : Notch signaling controls multiple steps of pancreatic differentiation. *Proc. Natl. Acad. Sci. USA.* 2003;100:14920–14925. 10.1073/pnas.2436557100 14657333 PMC299853

[42] Pagliuca FW : Generation of functional human pancreatic β cells in vitro. *Cell.* 2014;159:428–439. 10.1016/j.cell.2014.09.040 25303535 PMC4617632

[43] Hogrebe NJ Augsornworawat P Maxwell KG : Targeting the cytoskeleton to direct pancreatic differentiation of human pluripotent stem cells. *Nat. Biotechnol.* 2020;38:460–470. 10.1038/s41587-020-0430-6 32094658 PMC7274216

[44] Breunig M : Modeling plasticity and dysplasia of pancreatic ductal organoids derived from human pluripotent stem cells. *Cell Stem Cell.* 2021;28:1105–1124.e19. 10.1016/j.stem.2021.03.005 33915078 PMC8461636

[45] Darrigrand J-F Isaacson A Spagnoli FM : Generation of human iPSC-derived pancreatic organoids to study pancreas development and disease. *Figshare.* 2025. 10.6084/m9.figshare.28559300.v4 PMC12314479 40756323

