# Supplementary material for: Generation of human iPSC-derived pancreatic organoids to study pancreas development and disease
Source: F1000Res. 2025 Jun 10;14:575. [Version 1] doi: 10.12688/f1000research.162496.1 (PMC12314479; doi:10.12688/f1000research.162496.1)
Supplement: Peer review history

# Reviewer report: Reviewer response for version 1

**DOI:** 10.5256/f1000research.178705.r391179

**Published:** 2025-08-04

**Authors:** Naoki Tanimizu

**Affiliations:** 1 The University of Tokyo, Tokyo, Tokyo, Japan

The authors established the culture method generating pancreatic exocrine organoids from human iPS cells. The manuscript provides enough information for readers to perform the experiment.

 Only question of the reviewer is “the robustness of the culture”. The authors described that 93.5% of organoids acquired a branched architecture (page 11). The authors described that high purity of pancreatic progenitor (PDX1+NKX6.1+ should be more than 60%) is crucial for generating exocrine organoids. Does it depend on the purity of pancreatic progenitors before starting suspension culture? If the purity does not reach 60%, is there any way to increase the purity? Is it necessary to check the purity before suspension culture? Is there any organoid containing duct cells or cells expressing duct markers after rotation culture?

 If the authors provide additional information, it is very helpful for readers when they try a similar experiment. 

 In addition, before indexing the manuscript, the reviewer asks the authors to check several minor points.

 Page 7

 There are, “300 RCF for 3min” and “100 g for 3min”.

 Use RCF or g.

 Page 7,the last line

 ultra-low attachment -> ULA ?

 The authors established the culture method generating pancreatic exocrine organoids from human iPS cells. The manuscript provides enough information for readers to perform the experiment.

 Only question of the reviewer is “the robustness of the culture”. The authors described that 93.5% of organoids acquired a branched architecture (page 11). The authors described that high purity of pancreatic progenitor (PDX1+NKX6.1+ should be more than 60%) is crucial for generating exocrine organoids. Does it depend on the purity of pancreatic progenitors before starting suspension culture? If the purity does not reach 60%, is there any way to increase the purity? Is it necessary to check the purity before suspension culture?

 If the authors provide additional information, it is very helpful for readers when they try a similar experiment.

 In addition, before publishing the manuscript, the reviewer asks the authors to check several minor points.

 Page 7

 There are, “300 RCF for 3min” and “100 g for 3min”.

 Use RCF or g.

 Page 7,the last line

 ultra-low attachment -> ULA ?

Is the rationale for developing the new method (or application) clearly explained?

Yes

Is the description of the method technically sound?

Yes

Are the conclusions about the method and its performance adequately supported by the findings presented in the article?

Yes

If any results are presented, are all the source data underlying the results available to ensure full reproducibility?

Yes

Are sufficient details provided to allow replication of the method development and its use by others?

Yes

Reviewer Expertise:

Cell biology

I confirm that I have read this submission and believe that I have an appropriate level of expertise to confirm that it is of an acceptable scientific standard, however I have significant reservations, as outlined above.

# Reviewer report: Reviewer response for version 1

**DOI:** 10.5256/f1000research.178705.r391180

**Published:** 2025-07-31

**Authors:** Mostafa Bakhti, Christos Karampelias

**Affiliations:** 1 Helmholtz Center Munich, Neuherberg, Germany; 2 Helmholtz Munich, Munich, Germany

In this work, the authors present a new method for generating acinar enriched organoids from human pluripotent stem cells. There is a timely need in the field for acinar cell organoid generation and this manuscript addresses that with a Matrigel-free method based on reaggregation of pancreatic progenitors and protocol optimization. The manuscript overall is written clearly, address a clear need in the field and the methods part allow the reproducibility of the method by other laboratories. Some minor comments on the work are:

 To improve reproducibility of the protocol:

 - D11 part: Could you please specify what is the anti-adherence solution?

 - D14 part: Do you have an estimate of how many clusters need to be pooled for a successful protocol?

 - Have the authors checked if the protocol is reproducible with other stem cell lines that can be mentioned in the text?

 - How was the protocol optimization performed? Are there any pre-tests that can be included in the manuscript?

 For the biological relevance of the results:

 - The characterization of the acinar could be slightly expanded as the amylase staining has high background and in the qPCR CPA1 does not change much between D14-D19 days. One could test the PTF1A or RBPJL expression, transcription factors associated with acinar cells to understand better acinar cell induction. Also branching would imply some form of ductal cell fate have the authors tested any mature ductal cell markers that can be reported? 

 - These two papers could be cited for completion with close relevance to the topic

 Zhao J, et al., 2024 [Ref 1] (budding morphogenesis in stem-cells) and Andersson-Rolf A, et a., 2024 [Ref 2] (exocrine induction from human embryonic organoids).

Is the rationale for developing the new method (or application) clearly explained?

Yes

Is the description of the method technically sound?

Yes

Are the conclusions about the method and its performance adequately supported by the findings presented in the article?

Yes

If any results are presented, are all the source data underlying the results available to ensure full reproducibility?

Yes

Are sufficient details provided to allow replication of the method development and its use by others?

Yes

Reviewer Expertise:

Pancreatic development, beta cell research

We confirm that we have read this submission and believe that we have an appropriate level of expertise to confirm that it is of an acceptable scientific standard, however we have significant reservations, as outlined above.

## References

[1] : PDX1+ cell budding morphogenesis in a stem cell-derived islet spheroid system. *Nature Communications* .2024;15(1) : 10.1038/s41467-024-50109-2 10.1038/s41467-024-50109-2 PMC1124652939003281

[2] : Long-term in vitro expansion of a human fetal pancreas stem cell that generates all three pancreatic cell lineages. *Cell* .2024;187(26) : 10.1016/j.cell.2024.10.044 7394-7413.e22 10.1016/j.cell.2024.10.044 39626658

# Reviewer report: Reviewer response for version 1

**DOI:** 10.5256/f1000research.178705.r391177

**Published:** 2025-07-31

**Authors:** Maria Cristina Nostro

**Affiliations:** 1 McEwen Stem Cell Institute, Toronto, Canada

This manuscript describes a method for generating hiPSC-derived pancreatic organoids that recapitulate the early stages of pancreatic development, including the segregation of tip and trunk domains. The approach is novel in that it enables the study of early acinar specification from pancreatic progenitor cells, potentially alongside ductal and endocrine differentiation—although the latter was not assessed in this study.

 A key strength of this strategy is the differentiation of organoids in suspension cultures, without the use of animal-derived matrices.

 The manuscript is well-written, and the inclusion of tables helps clearly convey the protocol adaptations used to generate the pancreatic organoids.

 To further strengthen the study, it would be helpful to include:

1. Cell counts to calculate organoid production yield.
2. Evidence that the protocol is effective with at least one additional hiPSC line.
3. Staining or RT-qPCR analysis for ductal, endocrine, and additional acinar markers to assess whether ductal cells and/or endocrine progenitors are already present in the organoids—even in the absence of specific inducers such as TGF-β inhibitors.

Is the rationale for developing the new method (or application) clearly explained?

Yes

Is the description of the method technically sound?

Yes

Are the conclusions about the method and its performance adequately supported by the findings presented in the article?

Yes

If any results are presented, are all the source data underlying the results available to ensure full reproducibility?

No source data required

Are sufficient details provided to allow replication of the method development and its use by others?

Yes

Reviewer Expertise:

Diabetes research, Regenerative medicine, Stem Cell biology, Pancreas development, hPSC differentiation.

I confirm that I have read this submission and believe that I have an appropriate level of expertise to confirm that it is of an acceptable scientific standard.

# Reviewer report: Reviewer response for version 1

**DOI:** 10.5256/f1000research.178705.r391175

**Published:** 2025-07-29

**Authors:** Ilse Rooman

**Affiliations:** 1 Universiteit Brussel, Brussels, Belgium

The manuscript by Darrigrand et al. presents an original and robust, Matrigel-free protocol for hiPSC derived pancreatic organoids that recapitulate branching and trunk/tip cell differentiation. In particular, their protocol includes a step of Aggrewell aggregation and specific cell culture supplemments that leads them to consistently obtain branched organoids exhibiting NKX6.1⁺ trunk and AMY⁺ tip domains, closely recapitulating in vivo morphogenesis and acinar differentiation.

 Overall, the study represents a significant advance in human pancreatic organoids as an experimental platfrom and will be of broad interest to the stem-cell and cancer-modeling communities.

 Minor comments:

 - First line of introduction, swap “blood glucose levels” and “digestion” as it would be more logical with the previous part of the sentence.

 - Introduction: “…dedifferentiation of acinar cells is linked to PDAC onset in 98% of cases”. This may have to be rephrased. Most evidence comes from mouse models. I don’t think it has been properly quantified in humans. Also mind that duct cells can also be at origin of PDAC.

 - Inclusion of additional ductal (e.g. SOX9, HNF1B) and endocrine (e.g. INS) markers could be informative

 - Fig 3c: N=1 shown. Yet, there is statistical analysis. Is this on technical or biological repeats? This should be clarified.

Is the rationale for developing the new method (or application) clearly explained?

Yes

Is the description of the method technically sound?

Yes

Are the conclusions about the method and its performance adequately supported by the findings presented in the article?

Yes

If any results are presented, are all the source data underlying the results available to ensure full reproducibility?

Yes

Are sufficient details provided to allow replication of the method development and its use by others?

Yes

Reviewer Expertise:

pancreatic exocrine cell biology, pancreatic cancer

I confirm that I have read this submission and believe that I have an appropriate level of expertise to confirm that it is of an acceptable scientific standard.

# Reviewer report: Reviewer response for version 1

**DOI:** 10.5256/f1000research.178705.r393060

**Published:** 2025-07-29

**Authors:** Christof Westenfelder

**Affiliations:** 1 Division of Nephrology, Department of Internal Medicine, University of Utah Medical Centers, Salt Lake City, UT, 84112, USA

Dr. Spagnoli and colleagues present a Methods Article in which the in vitro differentiation of human iPSCs into non-endocrine pancreatic 3-D organoids is described. 

 The relevance of this technology lies in the ability to study exocrine pancreas development and potentially diseases such as pancreatitis and pancreas cancer as well as in vitro drug screening. The paper does, however, not test or demonstrate the claimed translational utility of the developed protocol. The tested protocol is in part based on protocols developed by others. Its potential utility of the utilized protocol to investigate the endocrine system of the pancreas is not discussed. 

 The experimental steps are adequately described and will readily allow others to utilize this approach.

 The following points should be addressed to improve the clarity and utility of the presented protocols:

 1. Pls. provide explanations for all used abbreviations throughout the paper.

 2. Pls. discuss how teratoma/teratocarcinoma formation is monitored and prevented.

 3. Since FGF-7 and -10 are used, what angiogenic differentiation is seen?

 4. Was potential & partial endocrine differentiation

 ruled out?

 5. Does the absence of endocrine differentiation affect the exocrine differentiation. Pls. discuss.

Is the rationale for developing the new method (or application) clearly explained?

Partly

Is the description of the method technically sound?

Yes

Are the conclusions about the method and its performance adequately supported by the findings presented in the article?

Partly

If any results are presented, are all the source data underlying the results available to ensure full reproducibility?

Yes

Are sufficient details provided to allow replication of the method development and its use by others?

Yes

Reviewer Expertise:

Stem Cell Biology, Endocrinology, Regenerative Medicine

I confirm that I have read this submission and believe that I have an appropriate level of expertise to confirm that it is of an acceptable scientific standard, however I have significant reservations, as outlined above.
